# Cytosolic PEPCK deficiency caused by a novel homozygous frame-shift variant presenting as resolved hypoglycemia and acute liver failure at birth^☆^

**DOI:** 10.1016/j.ymgmr.2024.101175

**Published:** 2024-12-19

**Authors:** Daniel Burg, Gheona Altarescu, Stanley Korman, Eyal Shteyer, Dalit May

**Affiliations:** a Department of Military Medicine and "Tzameret," Faculty of Medicine, Hebrew University of Jerusalem, and Medical Corps, Israel Defense Forces, Jerusalem, Israel; b Medical Genetics Institute, Shaare Zedek Medical Center, Hebrew University Hadassah Medical School, Jerusalem, Israel; c Metabolic Unit, Ruth Rappaport Children's Hospital, Rambam Health Care Campus, Haifa, Israel; d The Juliet Keidan Institute of Pediatric Gastroenterology, Shaare Zedek Medical Center, Jerusalem, Israel; e Faculty of Medicine, The Hebrew University of Jerusalem, Jerusalem, Israel; f Medical Genetics Institute, Shaare Zedek Medical Center, Jerusalem, Israel; g Clalit Health Services, Jerusalem, Israel

## Abstract

Cytosolic phosphoenolpyruvate carboxykinase (PEPCK) is an enzyme encoded by the PCK1 gene and plays a rate limiting step in gluconeogenesis occurring mainly in the liver during prolonged fasting. Biallelic deficiency of this enzyme results in a rare inborn error of metabolism disorder (OMIM # 261680). The main clinical and laboratory manifestations include fasting hypoglycemia and lactic acidosis with urinary excretion of Tricarboxylic Acid (TCA) cycles metabolites, particularly fumarate. The initial presentation varies between individuals in terms of age at initial presentation and clinical manifestations, however clinical information is lacking as it was diagnosed so far in less than 30 patients with a total of 6 different mutations which are all either missense or splice variants. We describe the first homozygous frame-shift mutation in the PCK1 gene, leading to cytosolic PEPCK deficiency. This resulted in transient hypoglycemia and acute liver failure with extreme hyperferritinemia (>40,000 ng/ml) during the first days of life. This severe very early-onset presentation that was not described earlier expands our clinical and genetic spectrum of this rare metabolic disorder.

## 1 Background

**Fig. 1:**
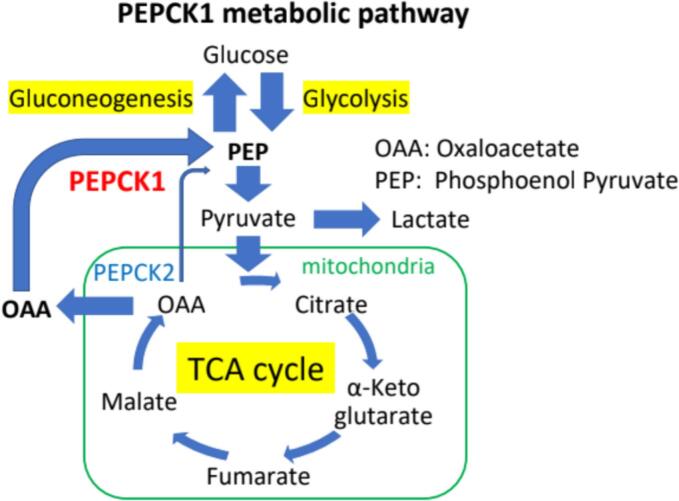
The figure illustrates the role of PEPCK1 in gluconeogenesis, converting oxaloacetate (OAA) to phosphoenolpyruvate (PEP) in the cytosol. The figure also shows the role of PEPCK2, the mitochondrial phosphoenolpyruvate carboxykinase, converting OAA that is generated in the tricarboxylic acid (TCA) cycle in the mitochondrial matrix, linking the TCA cycle to gluconeogenesis. Key metabolites such as pyruvate, lactate, and citrate are labeled, indicating their roles in these metabolic pathways.

Cytosolic phosphoenolpyruvate carboxykinase (PEPCK1) is an enzyme that plays a rate limiting step in gluconeogenesis occurring mainly in the liver during prolonged fasting (Fig. 1). In addition to converting oxaloacetate to phosphoenolpyruvate, PEPCK1 is involved in additional metabolic pathways including replenishing TCA cycle intermediates, facilitating fatty acid and triglyceride cycling and contributing to cancer cell growth and survival [[1], [2], [3], [4], [5]].

Another PEPCK isoenzyme involved in gluconeogenesis is the mitochondrial PEPCK (PEPCK2) active in the mitochondria of mammalian cells (Fig. 1).

PEPCK1 deficiency (OMIM # 261680) is a rare inborn error of metabolism disorder caused by biallelic loss of function of the cytosolic phosphoenolpyruvate carboxykinase (PEPCK1).

The main clinical and laboratory manifestations include fasting hypoglycemia and lactic acidosis with urinary excretion of TCA cycles metabolites, particularly fumarate [[6], [7], [8], [9]]. Other manifestations may include hepatic dysfunction [7,9] and hypoglycemic seizures [8,10,11]. The initial presentation varies between individuals and could be as early as the neonatal age, following an acute illness (such as gastroenteritis or febrile disease) during infancy or during stress later in life [8]. However, clinical information on this rare disease is lacking as it was diagnosed so far in less than 30 patients with a total of 6 different mutations which are all either missense or splice variants causing loss of function of the enzyme [[6], [7], [8], [9],^11^,^12^].

Here we describe the first homozygous frame-shift mutation in PCK1 gene manifested in transient hypoglycemia and acute liver failure with extreme ferritin levels (>40,000 ng/ml) that presented on the first days of life. This severe very early-onset presentation that was not described before, suggests that clinical severity and age at presentation are associated with the level of protein activity impairment, which is presumably profound in truncating mutations.

## 2 Clinical presentation

A neonate female, the second child of non-consanguineous parents from a Jewish Yemeni origin, was born following an uneventful pregnancy, except for a small gestational age. She was born after 41 weeks of pregnancy. Weight was 2.7Kg (8 % percentile). On her third day of life, due to reflux and weight loss hypoglycemia with glucose levels of 25 mg\dL was measured and she was transferred to the neonatal intensive care unit. Further blood workup revealed an acute liver failure with extreme hyper-ferritinemia of >40,000 ng/ml, high levels of ammonia and coagulopathy (Max level AST - 6445 (5–34) IU/L, ALT 6196 (9–52) IU/L, GGT 238 (12–43) IU/L, ALKP 211 (38–150) IU/L. Ammonia 200 (18–72) mcmol/L, PT 26.9 s and INR 2.26. Lactate mildly elevated 3.9 (0.7–2.1) mmol/L without acidosis (pH 7.4) (Table 1).

**Table 1:** Tests results showing acute liver failure on day 3 after birth that improved. Hospitalization at age 7 m showed - hypoglycemia and metabolic acidosis. AST- Aspartate Aminotransferase, ALT - Alanine Aminotransferase, INR -.

test	units	normal	max - neonatal	discharge - neonatal	max - age 7 m
Glucose	mg/dL	65–125	25	72	30
Alkaline Phosphatase	IU/L	38–150	211	186	654
activated AST (GOT)	IU/L	14–36	6445	55	179
activated ALT (GPT)	IU/L	9–52	6196	83	241
GGT	IU/L	12–43	87	238	14
Ferritin	ng/ml	11–205	>40,000	1254	200
LDH	IU/L	125–220	8061	382	338
Ammonia	mcmol/L	18–72	200		54
INR	INR	0.83–1.16	2.36	0.98	1.22
Triglycerides	mg/dL	35–160	106		298
pH		7.35–7.45	7.399	7.46	7.327
Lactate	mmol/L	0.5–2.2	3.9		4.5
HCO3-	mmol/L		20.9	29.5	14.1
blood beta-ketones	mmol/L	0.6–1.5			6.3

Otherwise she had no symptoms of infection and was neurologically intact, albeit a bit lethargic.

She was treated with dextrose and was further evaluated for acute neonatal liver failure. Liver imaging revealed normal US. MRI showed a mild degree of liver iron deposition. Neonatal metabolic screen and workup for infections came back negative. She was treated with IVIg in suspicion of Gestational alloimmune liver disease (neonatal hemochromatosis) and was discharged on day 16 of life with mild elevation of liver function tests (Table 1) for further outpatient follow-up and investigation.

Following neonatal discharge, she had a normal early infancy development and uneventful months, although a minimal elevation of liver enzymes persisted. At the age of 7 months she was admitted to the hospital with 3 days of fever, vomiting and apathy. On initial workup severe hypoglycemia (glucose level of 21 mg/dL) and metabolic acidosis with elevated lactate and blood ketone were detected (Table 1). Liver US showed enlarged liver with increased parenchymal echogenicity due to fatty infiltration.

The patient underwentadditional metabolic evaluations and genetic testing, including exome sequencing, that revealed a novel homozygous deletion in the PCK1 gene. The deletion of C at position 978 (chr20:56139240, hg19) results in frameshift of the open reading frame culminating in termination 15 amino-acids downstream. The deletion is located in an exon that is present in both known transcripts of the gene. In the main canonical longer transcript it is located in exon 7 (out of 10).

This variant was not previously reported (GnomAD). According to ACMG guidelines, this variant is classified as **pathogenic** (PSV1, PM2, PP1, PP4).

Metabolic test results further supported the exome finding of PEPCK1 deficiency and a defect in gluconeogenesis: hypoglycemia with ketotic and lactic acidosis (Table 1), massive urine secretion of tricarboxylic acid (TCA) cycle metabolites - in particularly fumarate but also - dicarboxylic acids, Glutaric acid and alpha-hydroxyglutaric acid as previously described [8,9]. In addition, ketonuria with high levels of 3-hydroxybutyrate was identified. High levels of Glutamine [6,8], Glutamic acid and Aspartic acids have also been observed. Triglycerides levels were also elevated (298 mg/dL, Table 1) due to fatty acid oxidation impairment [8].

The patient was discharged with instructions to avoid depletion of glycogen storage and provide glucose at times of acute illness. Glucose monitoring at night was also recommended. In a follow-up visit two weeks later - blood tests were normal except a minimal elevation in AST and ALT levels (60 and 56 IU/L, respectively, ferritin levels were normal (108 ng/ml) and so were triglycerides levels (44 mg/ml). urine showed persisting high levels of Krebs cycle metabolites without ketonuria and slightly elevated levels of Glutamic acid (171μmol/L, normal 1–150μmol/L). A follow-up visit at the age 15 month revealed that the patient was clinically stable. Apart from one episode of severe hypoglycemia (home glucose levels 30 mg/dL) in the morning after an acute illness and a small amount of intake in the prior evening; morning glucose levels were > 70 mg/dL. Body measurements at the age of 15 months: weight 9.5 kg (21 % percentile), height 76.5 cm (44 % percentile). Clinically she was very well and a minimal motor developmental delay was observed.

Genetic counseling and Family planning: Parental testing revealed both parents as healthy carriers of the pathogenic variant. The family was counseled on reproductive options, including prenatal diagnosis and preimplantation genetic testing (PGT). However, they did not pursue these options. The family recently had a new daughter delivered at our center. The newborn was tested for the mutation and confirmed to be a non-carrier.

## 3 Discussion and conclusion

PEPCK1 deficiency is a rare disorder of metabolism that was nearly impossible to diagnose at the pre-exome sequencing era [13,14].

The differential diagnosis for PEPCK1 deficiency includes metabolic and endocrinological disorders that can cause hypoglycemia, hepatic dysfunction, and elevated tricarboxylic acid intermediates in urinary organic acid analysis, e.g. other disorders of gluconeogenesis, glycogen storage diseases, fatty acid oxidation defects, and certain endocrine disorders like congenital hyperinsulinism.

So far, few reports have described the clinical presentation and course of this disease. Here we present the first case of PEPCK1 deficiency due to novel homozygous frame-shift mutation in PCK1 resulting in a unique presentation of severe early neonatal liver failure with extreme hyper-ferritinemia on the third day of life. The hepatic dysfunction and hypoglycemia nearly resolved completely upon dextrose administration and definite diagnosis was achieved during hospitalization at the age of 7 months when the patient was admitted due to acute illness. In our case, the differential diagnoses initially included neonatal hemochromatosis, fatty acid oxidation disorders and later glycogen storage diseases.

Whole exome sequencing showed a homozygous deletion of 1 bp in position 978 in the PCK1 gene causing a shift of the open reading frame and translation termination 15 amino-acids downstream. PEPCK-1 loss of function impedes cytosolic gluconeogenesis, and further biochemical and metabolic tests confirm the genetic diagnosis: hypoglycemia, liver dysfunction with fatty infiltration, lactic and ketoacidosis, tricarboxylic acid cycle metabolites in urine (particularly fumarate) and ketonuria. The rapid resolution following glucose level normalization further supports the diagnosis.

The loss of the cytosolic form of the phosphoenolpyruvate carboxykinase (PEPCK1) is thought to be partially replaced by the mitochondrial isoenzyme that contributes to hepatic gluconeogenesis [15] and therefore could explain the fact that this severe mutation in PCK1 is not fatal.

While inherited metabolic diseases are a well documented cause of neonatal liver failure [16] and variable levels of liver dysfunction were described in PEPCK1 deficiency [6,7,12], extreme hyper-ferritinemia was not previously described in this setting, and was very little investigated in the pediatric population [17]. Ferritin is mainly stored in hepatocytes and in macrophages. Therefore, hyperferritinemia that is unrelated to iron overload could be the result of a few conditions such as liver damage, inflammatory and infectious diseases [18,19]. Here, the level of ferritin exceeds the level of liver damage as reflected in other liver function tests (Table 1). We hypothesize that those levels could also represent the metabolic cross-talk between iron and glucose metabolism in the liver [20,21].

Finally, this case expands the clinical and genetic spectrum of this rare metabolic disorder and calls for more understanding on the potential role of the mitochondrial isoenzyme (PEPCK2) in hepatic gluconeogenesis by examining its cellular localization and expression levels in PCK1-deficient liver cells. Additional research could investigate PEPCK2 activity under physiological conditions as well as during metabolic stress. In addition we suggest that PEPCK deficiency should be suspected in the differential diagnosis of liver failure with extreme hyperferritinemia presenting at birth.

This case report was conducted following ethical guidelines, with written informed consent obtained from the patient's parents for publication of clinical and genetic data.

## CRediT authorship contribution statement

**Daniel Burg:** Writing – review & editing, Writing – original draft, Visualization. **Gheona Altarescu:** Writing – review & editing, Writing – original draft. **Stanley Korman:** Writing – review & editing, Writing – original draft, Resources. **Eyal Shteyer:** Writing – review & editing, Writing – original draft, Visualization, Resources. **Dalit May:** Writing – review & editing, Writing – original draft, Visualization, Validation, Resources.

## Declaration of competing interest

None.

## Data Availability

No data was used for the research described in the article.
